# Arrhythmogenic Cardiomyopathy: Diagnosis, Evolution, Risk Stratification and Pediatric Population—Where Are We? †

**DOI:** 10.3390/jcdd9040098

**Published:** 2022-03-27

**Authors:** Marianna Cicenia, Fabrizio Drago

**Affiliations:** Pediatric Cardiology and Arrhythmia/Syncope Complex Unit, Bambino Gesù Children’s Hospital, IRCCS, 00146 Rome, Italy; marianna.cicenia@opbg.net

**Keywords:** arrhythmogenic cardiomyopathy, children, premature ventricular contractions, sudden cardiac death, heart failure

## Abstract

Arrhythmogenic cardiomyopathy (ACM) is a cardiomyopathy characterized by the occurrence of a high risk of life-threatening ventricular arrhythmias and sudden cardiac death even at presentation. Diagnosis, evolution and outcomes in adults have been extensively reported, but little data in pediatric population are available. Risk stratification in this particular setting is still a matter of debate and new risk factors are needed in a model of an ever more “individualized medicine”.

## 1. Definition and Epidemiology

Arrhythmogenic cardiomyopathy (ACM) is a rare cardiac condition characterized by fibrous or fibro-fatty infiltration of the myocardium, leading to arrhythmias and progressive cardiac dysfunction. It is genetically determined and more than 50% of cases harbor variants in desmosomal genes, less commonly non-desmosomal ones.

Adult prevalence is estimated to be 1:5000, but the specific pediatric prevalence is not yet known, because only limited cohorts of patients or single cases have been reported in the literature [1].

Despite initially considered a disease exclusively affecting the right ventricle (RV), from the beginning of the 2000s, the concept of an analogous involvement of the left ventricle (LV) became apparent and the original term of arrhythmogenic right ventricular dysplasia (ARVD) was replaced by ACM. In particular Sen-Chowdrry et al. described a “classic” ARVC characterized by RV dominance, a “biventricular” pattern defined by parallel involvement of both ventricles and an LV dominant form with an extensive LV involvement and a “mirror-like” ARVD phenotypic presentation [2].

Myocardial loss and fibrous or fibro-fatty substitution with a subepicardialmid-mural distribution or with a transmural involvement in the absence of coronary artery disease has always been the diagnostic element for ACM [3].

**Figure 1:**
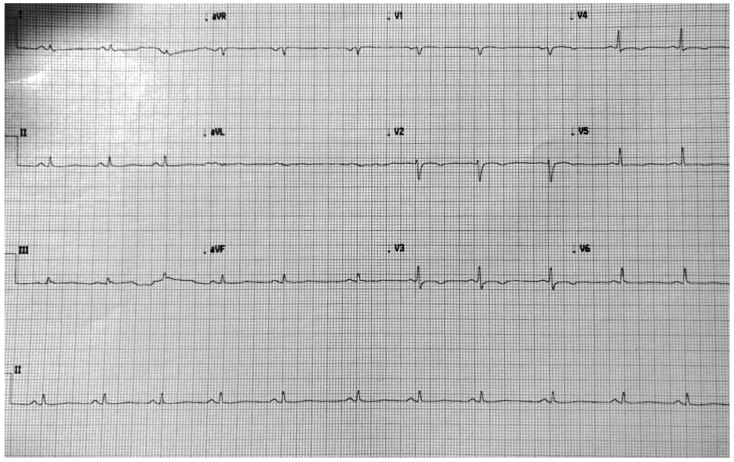
ECG showing diffuse repolarization abnormalities ad low voltages in limb leads.

Electrocardiographic (ECG) abnormalities (T wave inversion in right precordial leads or to the other leads, delayed S-wave upstroke in right precordial leads, right bundle branch block, low voltages in limb leads) and ventricular arrhythmias (isolated premature ventricular contractions, non-sustained or sustained ventricular tachycardias) are the expression of these histologic changes and can precede the structural phenotypic alterations (Figure 1).

ACM usually manifests between the second and fourth decade of life and rarely occurs during adolescence. Early symptoms include palpitations and syncope; sudden cardiac death can sometimes be the first manifestation of the disease, while heart failure is generally a sign of disease progression.

Notably, strenuous exercise can act as a phenotypic modifier and be the trigger for malignant arrhythmias and SCD as well [4].

Finally, LV dominant forms should be differentiated by other “phenocopies”, such as neuromuscular diseases with cardiac involvement, myocarditis, sarocoidosis and dilated cardiomyopathies.

In these cases, pathogenic or likely pathogenic variants of ACM-related genes allow the differentiation from LV-dominant ACM to the other phenocopies [5].

Differently, the Heart Rhythm Society (HRS), in 2019, included in the definition of ACM a broad spectrum of heart muscles disorders (not secondary to ischemia, hypertension or valvular disease) with a systemic, inflammatory, infectious or genetic cause, but with an elevated arrhythmic risk as a common denominator. This definition differs from the more commonly used in which ACM is a distinct condition characterized by typical structural, morpho-functional, histological, phenotypic and genetic features [6].

## 2. Diagnostic Criteria

Regarding the diagnosis of this heart disease, initially, the 1994 original Task Force criteria showed limitations concerning the lack of quantitative parameters, especially for grading RV dilatation/dysfunction and the amount of fibrosis at histology. Moreover, the arrhythmic aspects were considered “minor” criteria [7]. 

The 2010 revised International Task Force criteria (2010 ITF), instead, classified the ACM in “definite”, “borderline” and “possible” according to the number of satisfied criteria. Consequentially, these criteria improved the sensitivity of the previous ones giving more quantitative parameters and more consideration of the electrocardiographic and arrhythmic aspects. Notably, the identification of a pathogenic/likely pathogenic genetic variant was listed as a “major” criterion [8].

Very recently, the “Padua criteria” have updated the old criteria introducing the concept of the existence of forms limited to LV and the importance of fibrosis detected at cardiac magnetic resonance (CMR) and including three different phenotypic variants: dominant-right variant, dominant-left variant and biventricular variant. 

In these innovative criteria, the most important novelties have been: (1) the introduction of late gadolinium enhancement (LGE) at CMR as a major criterion; (2) a more important consideration of right ventricular dilation/dysfunction regardless of severity and of isolated regional abnormalities of left ventricular wall motility (minor criterion) in order to reflect the segmental nature of fibro-adipose substitution; (3) the importance of isolated PVCs not only in terms of absolute number (>500/24 h), but also in terms of morphology. Moreover, fibrous replacement at endomyocardial biopsy is no longer differentiated between major and minor criterion but is a major one, epsilon wave detection at ECG is a minor criterion and late potentials at signal-averaged ECG are no longer considered due to the low diagnostic accuracy.

Notably, the presence of at least one morpho-functional and/or structural major or minor criterion is essential to make diagnosis of ACM and the identification of a pathogenic or likely pathogenic ACM-causing gene mutation is mandatory to reach the diagnosis of “LV dominant” form. These clues give more specificity in the differentiation of ACM from other types of cardiomyopathies (CMPs) or arrhythmias (i.e., idiopathic ventricular tachycardias) [9,10].

## 3. Natural History

Sen-Chawdhry et al. reported four different and progressive stages of the disease evolution [11]. 

The early “concealed phase”, also called “hot phase”, is characterized by recurrent myocarditis-like episodes with preserved ventricular morphology and function. 

In this phase, the risk of sudden cardiac death (SCD) due to life-threatening arrhythmias exists, potentially being the first manifestation of the disease. These recurrent episodes and the inflammatory process may lead to disease progression. However, it is not well known if the inflammation is the cause of this progression or a reactive phenomenon to myocytes apoptosis in genetically predisposed individuals [12].

Subsequent stages are the consequence of fibrous (-fatty) myocytes replacement from the subepicardial to the subendocardial layers. The initial limited extension of the scar is responsible of the “overt phase” manifesting with electrical disorders. Only later, the progression of transmural fibrosis causes myocardial thinning, regional wall motion abnormalities and ventricular dysfunction [13].

## 4. Genetic Background

ACM is genetically determined and transmitted as an autosomal dominant trait, with incomplete penetrance and variable expressivity. Rarely an autosomal recessive transmission is involved in Naxos and Carvajal syndromes presenting with woolly hair palmoplantar keratoderma, nail dystrophy, dental anomalies, pemphigus-like vesicular lesions on palms, soles and knees, erosion and ulcers in perioral and sacral areas or hands and legs dorsal surfaces [14].

In at least 50% of patients, pathogenic or likely pathogenic variants (P/LP) (following the American College of Medical Genetics guidelines) of desmosomal genes are detected, even if non-desmosomal ones can also be involved. Furthermore, the prevalence of LP/P variants in young affected patients is reported to be significantly higher than adults in more than one cohort. 

Desmosomes are responsible for cell-to-cell adhesion and are part of a structure called intercalated discs (IDs). IDs are composed of adherent junctions, gap junctions and ion channels, which interact together and are responsible of the electrical, matabolic and structural properties of the cardiomyocytes. 

On the other hand, desmosomes are linked to the intermediate filaments of the cytoskeleton to guarantee structural stability and integrity against mechanical stress.

Mutation of desmosomal proteins, such as plakoglobin (*JUP*), plakophilin-2 (*PKP2*), desmoplakin (*DSP*), desmoglein (*DSG*) and desmocollin (*DSC2*), are historically considered the most involved in ACM pathogenesis.

Mutations in non-desmosomal proteins, such as laminin A/C (*LMNA/C*), desmin (*DES*), filamin C (*FLNC*), transmembrane protein 43 (*TMEM43*), ryanodine receptor-2 (*RyR2*), phos-pholamban (*PLN*) and transforming growth factor-3 (*TGFβ*), have also been considered as causative of ACM especially in biventricular and left-dominant forms. Genes encoding for adherent junctional proteins such as α-T-catenin (*CTNNA3*) and N-cadherin (*CDH2*) are also reported to be relevant for the pathogenesis, as they are essential for the cardiomyocytes interconnections and their alteration result in a common pathway consisting on the one hand of cells death and scarring and on the other hand of electrical disturbances and ions currents alterations. 

Despite these considerations and the numerous ACM related genes proposed, a variable and uncertain evidence of association has emerged over time. 

Recently, a group of experts reappraised 26 ACM genes reported in the literature and found that only 8 genes have a definite (*PKP2, DSP, DSG2, DSC2, JUP, TMEM43*) or moderate (*PLN, DES*) evidence for causing ACM.

RYR2, despite previously reported in association with ACM, was disqualified as an ACM-causative gene due to contradictory evidence and because it proved to be associated to catecholaminergic polymorphic ventricular tachycardia (CPVT) rather than ACM. 

All the other genes that were mentioned in various studies over several years as associated to ACM have a limited or no evidence of causality as well, and need more characterization over time. 

Since the identification of a LP/P variant is a “major criterion” of ACM diagnosis, an incorrect gene–disease association may lead to a misleading over-diagnosis, missing the correct diagnosis as well, and an incorrect familial screening and management. 

Thus, according to these authors, currently, only the previous mentioned variants (*PKP2*, *DSP*, *DSG2*, *DSC2*, *JUP*, *TMEM43*, *PLN*, *DES*) should be considered as “major criteria” in the diagnosis of ACM. 

LP/P variants in other genes may play a co-causative role and have a deleterious effect on the progression and on the electrical instability of the disease but they cannot be considered as diagnostic criteria. In the same way, variants of unknown significance (*VUS*) often detected in the genetic screening/testing are not to be considered as “major criteria” but their eventual role in the disease cannot be excluded and the interpretation of the results by an expert in cardiogenetics and an integrated approach with the cardiologists is of paramount importance to help to translate the genetic findings to the clinical practice. Moreover, in the future, the eventual reclassification of current *VUS* variants will eventually lead to redefine the diagnosis of “suspected” ACMs which do not yet reach the criteria [5,13,14,15,16,17].

## 5. Risk Stratification

Once the diagnosis of ACM is established, the most important issue is the risk stratification for SCD and the decision to implant an ICD.

In 2015, Corrado, considering “major” and “minor” risk factors, proposed an algorithm to classify the patients in three different categories of arrhythmic risk: high, moderate and low. Consequently, ICD implantation can be indicated in class I, IIa, IIb or not indicated. In detail the major risk factors for risk stratification are the experienced cardiac arrest or life-threatening arrhythmias, non-sustained VT (NSVT), the grade of heart dysfunction (RV/LV moderate or severe heart dysfunction) and syncopal events. While the minor risk factors are more heterogeneous and include proband status, male gender, electrical instability (spontaneous VAs or induced at electrophysiological study), younger age, complex genotype and the extent of structural heart disease [1,18].

Among young patients, the most common presentation of SCD/VF was largely confirmed by Bhonsale et al., along with male sex as a risk factor in terms of SCD, symptoms and life-threatening arrhythmias and otherwise unrelated to the involvement of left ventricle and cardiac arrest [19]. 

More recently, two other algorithms for the prediction of life-threatening VAs have been proposed [20,21]. 

Cadrin-Tourigny et al., in 2019, aimed to create a prediction model of VAs and SCD in ACM patients. This necessity emerged from the fact that previous recommendations were based on expert opinions and provided only categorical classes of SCD risk in order to advise ICD implantation. They differently proposed an algorithm seeking to evaluate the SCD risk as a continuum variable using six already known risk predictor variables: sex, age, recent (<6 months) cardiac syncope, NSVT, number of PVCs on 24-h Holter monitoring, extent of T-wave inversion (TWI) on anterior and inferior leads, RV ejection fraction and LV ejection fraction. The primary outcome was the first sustained VA in patients with ACM diagnosis who had never been experienced a similar event before or with an ICD implanted for primary prevention. In this regard, sustained VA was considered as the occurrence of SCD, sustained VT, ventricular fibrillation/flutter and appropriate ICD intervention. This model was proven to accurately distinguish patients who will have VAs for those who will not, allowing an appropriate patient selection and avoiding inappropriate ICD implants and their considerable risk complications especially in young people. In detail this model resulted in a 20.6% reduction of ICD implantations compared to the current algorithm, with a higher net benefit of protection. Of note, this study, even though it was the largest one conducted before 2019 (528 patients), was limited by the higher prevalence of Caucasian patients, the higher prevalence of *PKP2* variants identified and the use of ICD shocks as surrogate of SCD, while a great proportion of VTs in ACM patients would have been self-limited in the absence of ICD therapy [20].

Subsequently, the same group aimed to specifically predict the risk of life-threatening VAs as a surrogate of SCD, to overcome the risk to overestimate VA cases, considering all the sustained VAs. This was supported by the concept that stable VAs, even if sustained, and the potentially fatal VAs do not underlie the same predictors. They aimed to create a new prediction model for potentially fatal VAs and SCD. In this regard they found that only four of the classically considered risk factors were predictive of life-threatening VAs: male sex, young age at presentation, high PVC burden and number of TWI at ECG. 

Interestingly, prior sustained VAs were not predictive of life-threatening arrhythmic events and this could help to not overestimate the SCD risk and the consequent inappropriate ICD implantation, neither the extent of functional impairment (RV and LV dysfunction) nor syncopal event. This apparently weird conclusion may be explained by the concept that an early electrical phase and electrical instability may lead to unstable VAs independently by the structural substrate [21].

Recently, Aquaro also proposed the presentation of the CMR phenotype for risk stratification [16]. In fact, tissue characterization and regional wall motion abnormalities of both ventricles were not previously considered in the risk score model by Cadrin-Tourigny et al. [20] These variables were included in the 5-year risk algorithm. 

Moreover, in this study, combined endpoints of SCD, appropriate ICD intervention and aborted cardiac arrest were considered. The results showed that the 5-year risk score was significantly higher in patients with LV involvement than those without. Therefore, left ventricular involvement should be considered a marker of worse prognosis and the risk of major events may be relevant even in the absence of impaired systolic function. On the other hand, the RV-lone phenotype has the lowest risk.

It was also supposed that the elevated risk in case of biventricular involvement is the result of RV disease progression, whereas LV dominant forms may be considered a different disease with different genotype and phenotype with less functional impairment. 

However, all these data enhance the importance of the CMR, as echocardiography may often be normal in these patients. 

Finally, the Cadrin-Tourigny risk score may be valid for lone RV presentation, but not if LV is involved. This may result in a redefinition of the 5-year risk score: in case of RV phenotype, the 5-year risk score should be used and ICD considered for a risk >15%, while in case of LV involvement, the average risk was >15% by default and ICD should be recommended [22]. Further studies are needed to validate applicability of these algorithms in the guidelines.

It is essential to remember that the arrhythmic risk is always present over the time, for the progressivity of the disease itself, and can change rapidly and becoming higher, so the risk assessment must be done periodically, especially at young age [23].

Heart failure (HF) is another complication occurring in the advanced stages of the disease and it requires treatment with traditional pharmacological therapies or advanced mechanical support and heart transplant (HT) [15]. 

Its prevalence in ACM varies according to the different cohort characteristics and the different HF definition criteria, historically mainly focused on LV dysfunction. 

Gilotra et al., in 2017, described the prevalence, manifestations and predictors of HF in a large cohort of ACM patients. HF was defined in the presence of at least one sign or symptom of HF in presence of ACM diagnosis. In this study, despite classically considered a rare event in this contest, HF was found to be more prevalent than previously reported. Possibly, this discrepancy could be attributable to the consideration of symptoms and signs of HF and not only the volume overload and ventricular dysfunction, and the exclusion of the at-risk family members (not yet affected siblings). In fact, signs or symptoms may be present even in the absence of ventricular dilatation or dysfunction. 

Of note, despite men having a worse prognosis in this disease, women had a higher risk of HF in this study, not based on the presence of LV involvement. Moreover, heart failure was not significantly influenced by the genotype, but all the patients with complex genotype had symptomatic HF. Notably, patients referred for HT were more likely to have RV failure or refractory ventricular arrhythmias, rather than classic LV dysfunction.

Given the high life-threatening arrhythmic risk, this condition remains an electrophysiologist managed disease, however, with the higher efficacy in SCD prevention, a longer life expectancy of these patients is wished with subsequent higher incidence of HF and needing of HT in the future [24].

Beyond the phenotypic aspects, the knowledge of the genetic background has led to a more comprehensive assessment of the disease and of the different presentations. 

The largest study, which sought to determine the impact of the genotype on the ACM course and outcome, was conducted by Bhonsale et al., in 2015.

Carriers of more than one mutation (digenic or compound heterozygous variants) were found to have worse outcome, earlier onset of symptoms and arrhythmias and a greater risk of LV dysfunction and heart failure (HF) than those carrying a single mutation. Among the latter, this study demonstrated that the arrhythmic risk is always present and not related to specific ACM associated genes; conversely, specific genes such as *DSP* and *PLN* confer a higher risk of LV involvement and HF [19].

Further studies were focused to the genetic aspects. For example, *DSP LP/P variants*, p. S358L in the gene *TMEM43* and *PLN*-pArg14, have been associated to a worse prognosis and elevated risk of SCD and a ring-like pattern of subepicardial left ventricle (LV) fibrosis can be identified and gives a high risk of SCD in *FLNC* and *DES* mutation carriers.

All these important features create the basis for an “individualized” medicine and risk stratification, as already proposed by Verstralen et al. for the carriers of the *PLN*-pArg14 [13,15,25,26].

Even a “*DSP* cardiomyopathy” has been identified as a kind of distinct form of ACM characterized by myocardial inflammation, fibrosis and LV systolic dysfunction in the later stages, predisposing to ventricular arrhythmias [27].

All these data reinforce the importance of personalized risk stratification in this particular patient setting and the importance of the phenotypic and genetic family screening once a member has the ACM diagnosis received.

## 6. ACM in the Pediatric Population

Regarding the ACM presentation in the pediatric population, there are only a few studies with a limited number of patients and relatively short follow-up.

In 1995, Daliento first attempted to compare young vs adult patients with ACM. This author reported that in young patients with ACM there is a greater amount of fibrosis detected on endomyocardial biopsy (EBM) and a higher incidence of ventricular fibrillation (VF) and SCD than in adults [28].

In 2011, Bauce analyzed a cohort of pediatric patients affected by ACM carrying desmosomal gene variants. She confirmed the high risk of life-threatening VAs in the young and reported an important correlation between DSP mutation and a worse disease progression in terms of RV and LV dilatation/dysfunction, moving toward the concept that ACM is not only a RV disease [29]. These important findings were confirmed by Chungsomprasong, who reported the involvement of the LV in children and adolescents as a stronger predictor of adverse outcome including the need for heart transplantation [30].

In the same period, Te Riele, comparing ACM with pediatric-onset vs adult onset, reported that SCD can be more often the presenting symptoms in pediatric patients, whereas adults present more frequently with hemodynamically stable sustained VTs, but other features and outcomes, once ACM is diagnosed, were similar, in contrast to the most CMPs with a pediatric onset, where an early-onset is related to a more adverse clinical course. Moreover, in this cohort, children were more often gene mutation carriers [31].

This led to Deshpande et al. suggesting the necessity of a modification of the 2010 ITF criteria, especially for the pediatric patients, due to the possible underestimation of occurrence of ACM, since some criteria (i.e., ECG criteria) were less applicable in children [32].

The importance and accuracy of the CMR for the diagnosis of ACM have been validated by several studies, also in children and adolescents [33,34,35,36]. 

Another important feature of ACM in pediatric patients is the recurrent myocarditis-like episodes [37]. There is a “hot phase” of clinical presentation in children with more frequent chest pain episodes, higher values of troponin I/CPK and more edema/hyperemia at cardiac magnetic resonance (CMR) than in ACM adult patients [12,36].

Notably, Te Riele, did not find any similar presentation in his study. Possibly, this is due to the presence of a predominant *PKP2* variants in his pediatric cohort [31], compared to the other studies where *DSP* variants were well represented [12,36,37].

In this regard, the presence of *DSP* variants may lead to the “hot phase” presentation and life-threatening arrhythmias at the exordium, differently by the *PKP2* variants. Moreover, the inflammatory theory and the demonstration of serum pro-inflammatory cytokines have led to the attempt to target the inflammatory pathways as a new medical therapy. The discovery of serum anti-*DSG2*, anti-heart and anti-intercalated disk auto-antibodies supported also an autoimmunity hypothesis, but larger cohorts and prospective studies are needed to confirm this [31,38,39].

Heart failure, commonly considered an event occurring during the advanced stages, has a high prevalence among the pediatric cohort as described by Surget et al. In particular, in his study, heart failure was the first clinical manifestation in 37% of patients in the pre-puberty group (group 1), where the LV dominant and the biventricular involvement were the more prevalent forms and *DSP* variants were more frequently detected. Thirty-three percent of these patients underwent to HT or died during follow-up. Conversely, pediatric patients of post-pubertal age (group 2) had more ventricular tachycardias than the initial presentation, and the RV dominant form was the predominant phenotype together with the higher presence of *PKP2* variants [40].

The discrepancy between these findings and the previous ones could be explained by the younger age of the analyzed patients, confirming the potential variability of presentation of this disease in case of different age, sex and genetic background.

Very recently, Roudijk et al. described the clinical characteristics and outcomes of the largest pediatric cohort with ACM (probands and relatives). It has been confirmed that SCD is potentially the first manifestation of ACM in both pediatric probands and relatives, although ACM may be rare in children. Consequently, caution should be exercised if suspected, as life-threatening events can occur. In this regard, electrical characteristics in terms of ECG, PVC burden, complex PVC and CMR results are of fundamental importance for diagnosis and also for identifying disease progression [41]. However, despite being the largest pediatric cohort analyzed, ACM was diagnosed according to the 2010 Revised Task Force Criteria [8].

Differently, our group recently reported clinical and diagnostic features of a cohort of 21 pediatric patients affected by ACM, encompassing the Padua criteria and comparing them to the 2010 Revised Task Force Criteria eventually satisfied. Most patients of our cohort presented with PVCs, stable ventricular arrhythmias and fibrous myocardial substitution. Interestingly, the high prevalence of the PVCs with a left bundle branch block (LBB) morphology and inferior axis, coming from with right ventricular outflow tract (RVOT), in our patients may suggest that in pediatric patients, differently from the adult patients, this morphology should be considered with the same attention of the non-RVOT one.

Moreover, the extreme variability of the exercise testing in term of ventricular arrhythmias behavior could confirm the previous results by Sequeira et al., highlighting the impossibility to derive “definite conclusions” after this test in case of suspicion of ACM in pediatric setting [42]. In particular, this encourages not considering by default “benign PVCs” that disappear during exercise.

In our study, “Padua criteria” were proven to also be more accurate in this setting, since, if our patients had been analyzed according to the ITF criteria, there would have been an underestimated number of diagnoses. Moreover, in this our experience, CMR was confirmed to be the major diagnostic tool allowing a specific diagnosis in comparison with the control group of idiopathic VAs.

Notably, the arrhythmic aspect remained a major aspect of the disease, even if a minority of patients presented with aborted SCD or life-threatening arrhythmias. Furthermore, the electrophysiologic study (EPS) showed inducible VT in only a minority of patients, differently from the other studies, and electroanatomic mapping (EAM) showed high prevalence of low voltage areas with fragmented electrocardiograms [43,44].

## 7. Conclusions

ACM is an extremely heterogeneous disease due to the genetic background not always being detectable and the possible co-existence of inflammatory and autoimmune disorders, together with metabolic or external causes which act as epigenetic factors; moreover, the similitude to other “phenocopies” makes the diagnosis uncertain and equivocal in many cases, despite the presence of well-defined major and minor diagnostic criteria. In this setting, ACM may be considered a kind of syndrome with many possible interacting factors leading to similar or even different phenotypes.

The rarity of ACM presentation in pediatric age makes its characterization more difficult. Pediatric patients are more likely to experience SCD or aborted SCD. This may be the consequence of the more frequent myocarditis-like episodes and the consequent electrical instability in this setting. The first level screening especially in the pre-pubertal and pubertal age is very important as well as the careful evaluation of even the “simple” premature ventricular contractions to rule out the disease at the earlier stages, especially in case of symptoms or positive family history.

Even if in the past the “Old” diagnostic criteria have been advocated as not adequate for the pediatric age, due to some pitfalls such as the T-wave inversion in right precordial leads (which is normal in pre-pubertal age), the Padua criteria have been proven to also have a good diagnostic accuracy in this particular context.

Arrhythmic risk stratification remains the first aim once ACM is diagnosed. There is still no risk prediction model for risk stratification in the pediatric setting, but its conceptualization in the future should be aimed. At any age, due to the heterogeneity and variability of the disease presentation and the possible multiple underlying factors, the risk must be strictly individualized and the most updated knowledge (e.g., genotype, LV involvement, extensive fibrosis) must be taken into account together with the “older recommendations”. Moreover, the absolute risk could change during time in the individual cases and a periodical reassessing in warranted.

EPS and EAM can be useful for risk stratification, especially in the case of uncertain indication to ICD, but their specificity is still limited by the low reproducibility and technical variability.

## Data Availability

Not applicable.
